# Stent with vacuum therapy for treatment of colonic anastomotic leakage

**DOI:** 10.1055/a-2234-4462

**Published:** 2024-02-02

**Authors:** Kirill Basiliya, Erik J. van Helden, Myrthe de Jong, Indy Planting, Fabian Holman, Akin Inderson

**Affiliations:** 1 Department of Gastroenterology and Hepatology, Leiden University Medical Center, Leiden, Netherlands; 2 Department of Surgery, Leiden University Medical Center, Leiden, Netherlands

**Fig. 1:**
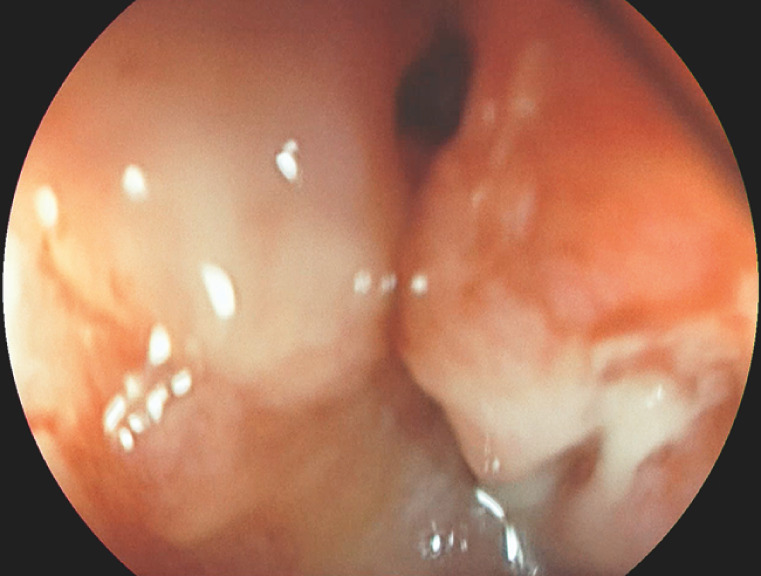
Anastomotic defect.

A 64-year-old patient with a history of severe biliary pancreatitis complicated by partial small-bowel resection and extensive adhesiolysis, and a sigmoid resection with colostomy formation due to diverticulitis, presented with an anastomotic leak and intra-abdominal collections after colostomy reversal ( Fig. 1 ). This case presented a challenge as there was no possibility of surgical correction, and thus, any endoscopic option would have to contend with ongoing fecal flow.

An over-the-scope clip was considered but the luminal diameter was too narrow to allow adequate positioning of the device. An Endo-SPONGE (B. Braun, Meslungen, Germany) was considered but rejected due to the inability to deviate the fecal flow. A colonic stent would provide defect cover but has a high risk of migration and would not stimulate wound healing. A VacStent GI (VacStent Medtech AG, Steinhausen, Switzerland) is a fully covered self-expanding metal stent covered with a sponge, which enables both sealing of the defect as well as local vacuum therapy, and given these characteristics it was decided to use the VacStent to achieve defect closure ( Video 1 ).

Successful treatment of anastomotic defect with VacStent (VacStent Medtech AG, Steinhausen, Switzerland).Video 1

**Fig. 2:**
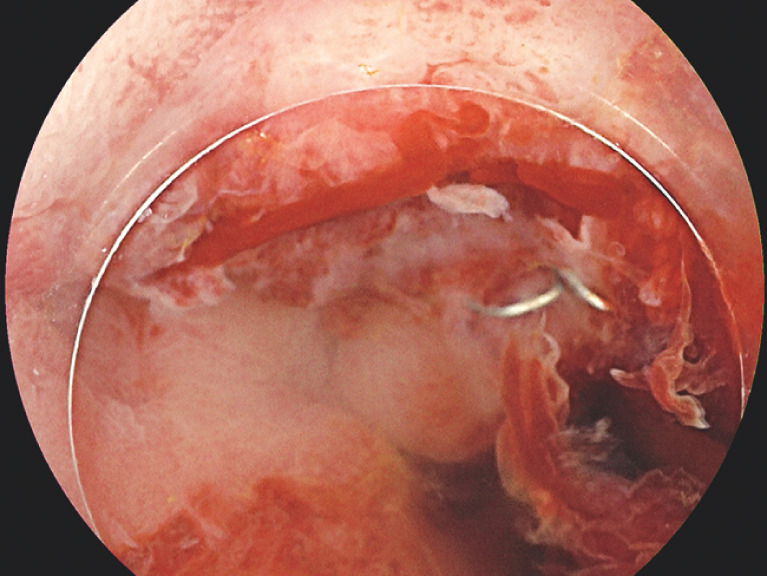
Complete defect closure after VacStent treatment (VacStent Medtech AG, Steinhausen, Switzerland).

The VacStent was developed to treat esophageal perforations and upper gastrointestinal anastomotic leakage but, to our best knowledge, has never been used in the colon. After 7 days of VacStent application, full defect closure was achieved while the patient continued to have daily bowel motions ( Fig. 2 ). This opens up an exciting novel endoscopic treatment option for colonic anastomotic defects and offers potentially significant advantages compared with existing endoscopic options, as it provides both defect closure as well as vacuum therapy, while allowing uninterrupted fecal flow and obviating the need for a temporary diverting colostomy.

Endoscopy_UCTN_Code_TTT_1AQ_2AG

